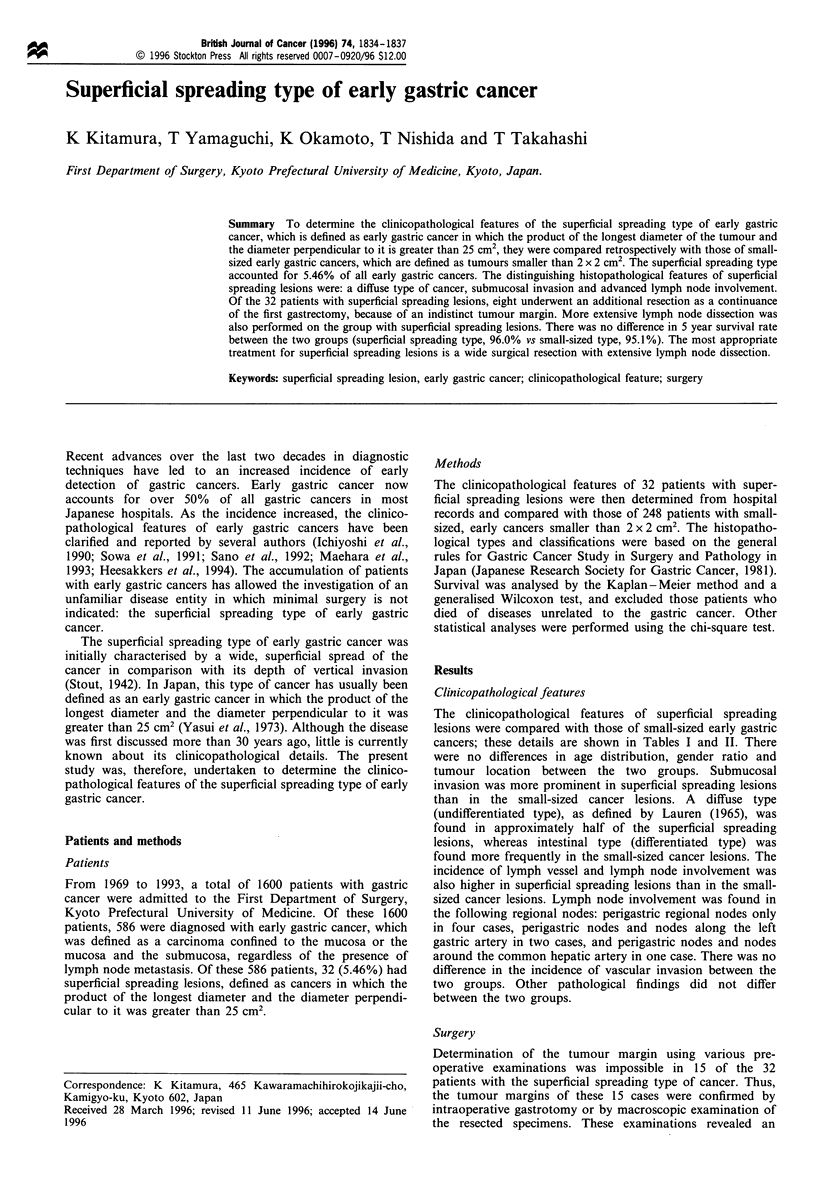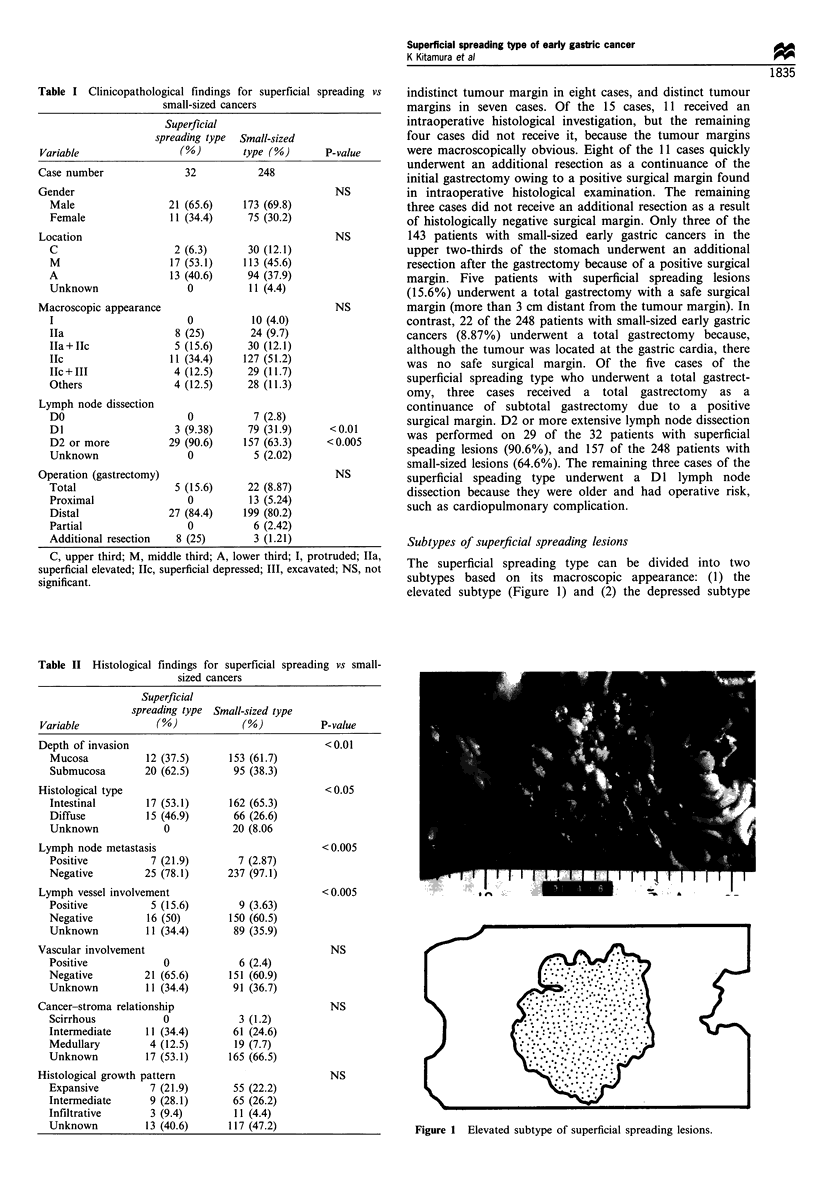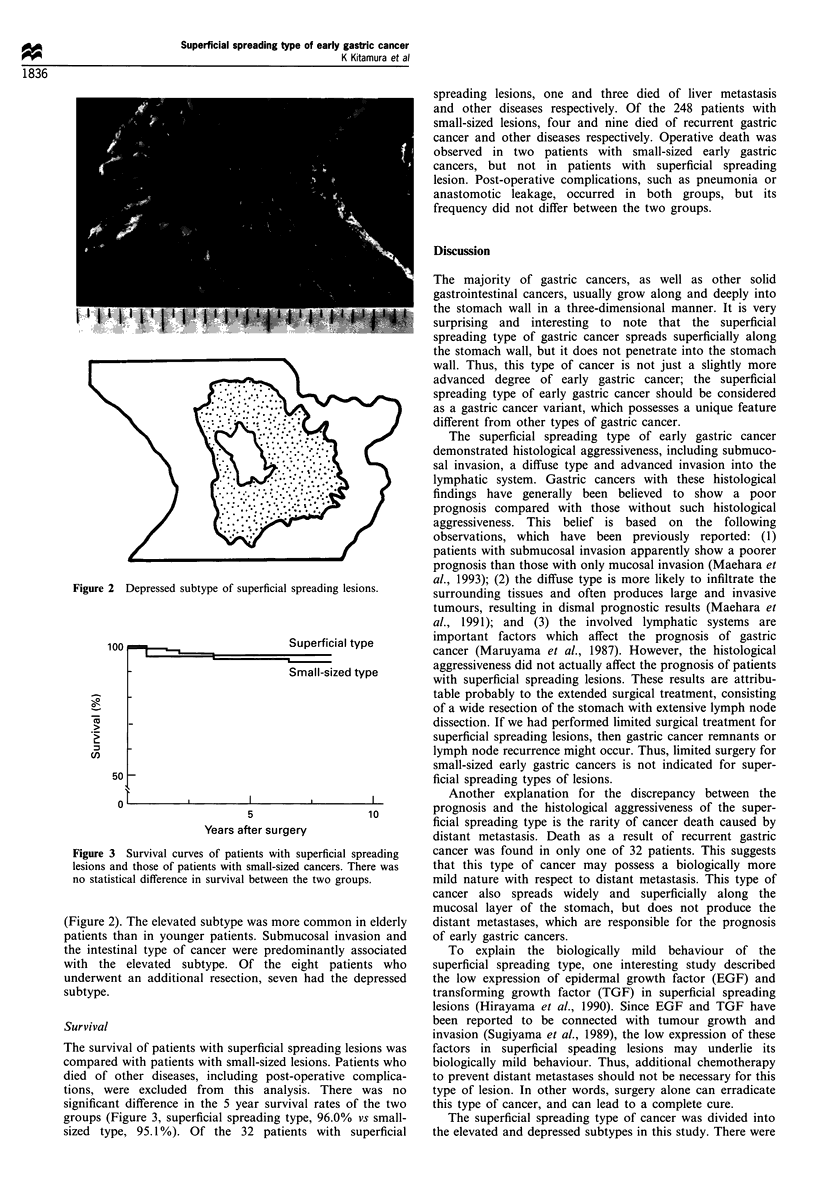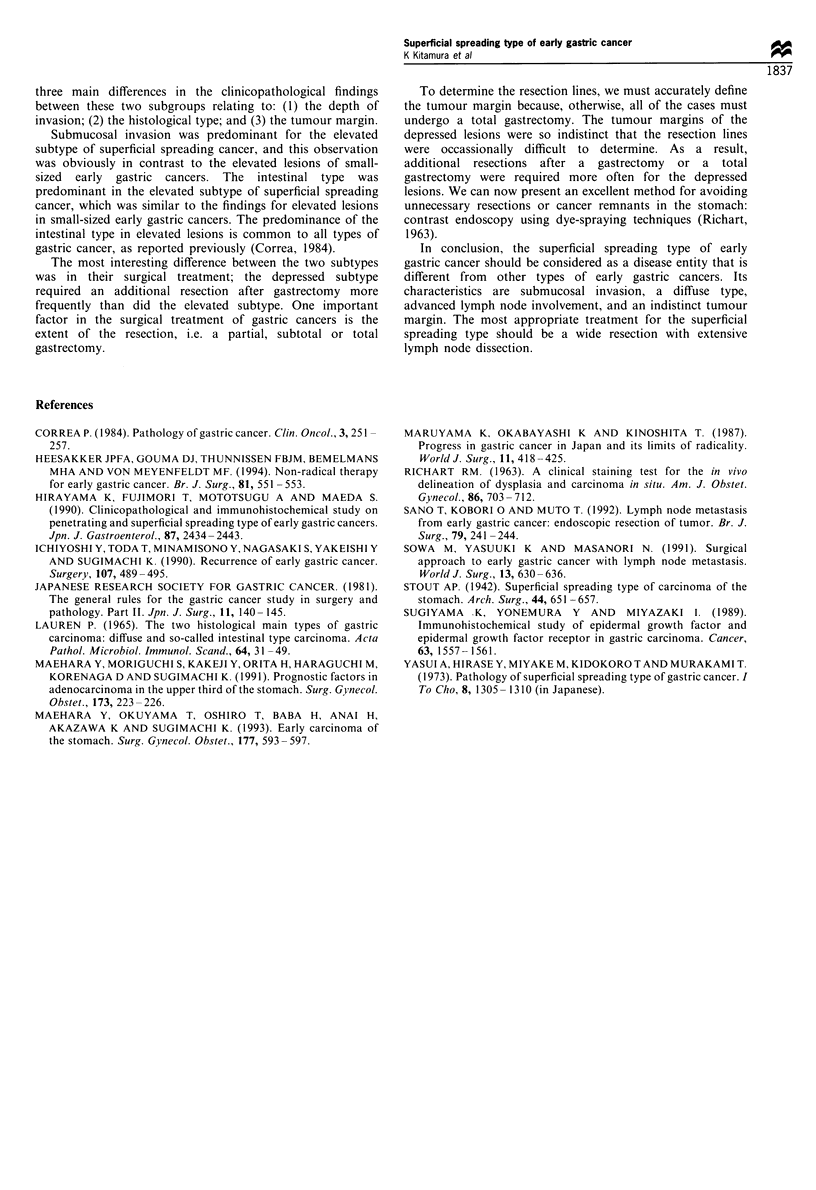# Superficial spreading type of early gastric cancer.

**DOI:** 10.1038/bjc.1996.639

**Published:** 1996-12

**Authors:** K. Kitamura, T. Yamaguchi, K. Okamoto, T. Nishida, T. Takahashi

**Affiliations:** First Department of Surgery, Kyoto Prefectural University of Medicine, Japan.

## Abstract

**Images:**


British Journal of Cancer (1996) 74, 1834-1837
© 1996 Stockton Press All rights reserved 0007-0920/96 $12.00

### Superficial spreading type of early gastric cancer

K Kitamura, T Yamaguchi, K Okamoto, T Nishida and T Takahashi

*First Department of Surgery, Kyoto Prefectural University of Medicine, Kyoto, Japan.*

**Summary** To determine the clinicopathological features of the superficial spreading type of early gastric cancer, which is defined as early gastric cancer in which the product of the longest diameter of the tumour and the diameter perpendicular to it is greater than 25 $cm^2$, they were compared retrospectively with those of small-sized early gastric cancers, which are defined as tumours smaller than $2 \times 2$ $cm^2$. The superficial spreading type accounted for 5.46% of all early gastric cancers. The distinguishing histopathological features of superficial spreading lesions were: a diffuse type of cancer, submucosal invasion and advanced lymph node involvement. Of the 32 patients with superficial spreading lesions, eight underwent an additional resection as a continuance of the first gastrectomy, because of an indistinct tumour margin. More extensive lymph node dissection was also performed on the group with superficial spreading lesions. There was no difference in 5 year survival rate between the two groups (superficial spreading type, 96.0% *vs* small-sized type, 95.1%). The most appropriate treatment for superficial spreading lesions is a wide surgical resection with extensive lymph node dissection.

**Keywords:** superficial spreading lesion, early gastric cancer; clinicopathological feature; surgery

Recent advances over the last two decades in diagnostic techniques have led to an increased incidence of early detection of gastric cancers. Early gastric cancer now accounts for over 50% of all gastric cancers in most Japanese hospitals. As the incidence increased, the clinicopathological features of early gastric cancers have been clarified and reported by several authors (Ichiyoshi *et al.*, 1990; Sowa *et al.*, 1991; Sano *et al.*, 1992; Maehara *et al.*, 1993; Heesakkers *et al.*, 1994). The accumulation of patients with early gastric cancers has allowed the investigation of an unfamiliar disease entity in which minimal surgery is not indicated: the superficial spreading type of early gastric cancer.

The superficial spreading type of early gastric cancer was initially characterised by a wide, superficial spread of the cancer in comparison with its depth of vertical invasion (Stout, 1942). In Japan, this type of cancer has usually been defined as an early gastric cancer in which the product of the longest diameter and the diameter perpendicular to it was greater than 25 $cm^2$ (Yasui *et al.*, 1973). Although the disease was first discussed more than 30 years ago, little is currently known about its clinicopathological details. The present study was, therefore, undertaken to determine the clinicopathological features of the superficial spreading type of early gastric cancer.

### Patients and methods

*Patients*

From 1969 to 1993, a total of 1600 patients with gastric cancer were admitted to the First Department of Surgery, Kyoto Prefectural University of Medicine. Of these 1600 patients, 586 were diagnosed with early gastric cancer, which was defined as a carcinoma confined to the mucosa or the mucosa and the submucosa, regardless of the presence of lymph node metastasis. Of these 586 patients, 32 (5.46%) had superficial spreading lesions, defined as cancers in which the product of the longest diameter and the diameter perpendicular to it was greater than 25 $cm^2$.

Correspondence: K Kitamura, 465 Kawaramachihirokojikajii-cho, Kamigyo-ku, Kyoto 602, Japan
Received 28 March 1996; revised 11 June 1996; accepted 14 June 1996

*Methods*

The clinicopathological features of 32 patients with superficial spreading lesions were then determined from hospital records and compared with those of 248 patients with small-sized, early cancers smaller than $2 \times 2$ $cm^2$. The histopathological types and classifications were based on the general rules for Gastric Cancer Study in Surgery and Pathology in Japan (Japanese Research Society for Gastric Cancer, 1981). Survival was analysed by the Kaplan–Meier method and a generalised Wilcoxon test, and excluded those patients who died of diseases unrelated to the gastric cancer. Other statistical analyses were performed using the chi-square test.

### Results

*Clinicopathological features*

The clinicopathological features of superficial spreading lesions were compared with those of small-sized early gastric cancers; these details are shown in Tables I and II. There were no differences in age distribution, gender ratio and tumour location between the two groups. Submucosal invasion was more prominent in superficial spreading lesions than in the small-sized cancer lesions. A diffuse type (undifferentiated type), as defined by Lauren (1965), was found in approximately half of the superficial spreading lesions, whereas intestinal type (differentiated type) was found more frequently in the small-sized cancer lesions. The incidence of lymph vessel and lymph node involvement was also higher in superficial spreading lesions than in the small-sized cancer lesions. Lymph node involvement was found in the following regional nodes: perigastric regional nodes only in four cases, perigastric nodes and nodes along the left gastric artery in two cases, and perigastric nodes and nodes around the common hepatic artery in one case. There was no difference in the incidence of vascular invasion between the two groups. Other pathological findings did not differ between the two groups.

*Surgery*

Determination of the tumour margin using various preoperative examinations was impossible in 15 of the 32 patients with the superficial spreading type of cancer. Thus, the tumour margins of these 15 cases were confirmed by intraoperative gastrotomy or by macroscopic examination of the resected specimens. These examinations revealed an

**Table I** Clinicopathological findings for superficial spreading *vs* small-sized cancers

| *Variable* | *Superficial spreading type (%)* | *Small-sized type (%)* | P-*value* |
|---|---|---|---|
| Case number | 32 | 248 | |
| Gender | | | NS |
| Male | 21 (65.6) | 173 (69.8) | |
| Female | 11 (34.4) | 75 (30.2) | |
| Location | | | NS |
| C | 2 (6.3) | 30 (12.1) | |
| M | 17 (53.1) | 113 (45.6) | |
| A | 13 (40.6) | 94 (37.9) | |
| Unknown | 0 | 11 (4.4) | |
| Macroscopic appearance | | | NS |
| I | 0 | 10 (4.0) | |
| IIa | 8 (25) | 24 (9.7) | |
| IIa + IIc | 5 (15.6) | 30 (12.1) | |
| IIc | 11 (34.4) | 127 (51.2) | |
| IIc + III | 4 (12.5) | 29 (11.7) | |
| Others | 4 (12.5) | 28 (11.3) | |
| Lymph node dissection | | | |
| D0 | 0 | 7 (2.8) | |
| D1 | 3 (9.38) | 79 (31.9) | < 0.01 |
| D2 or more | 29 (90.6) | 157 (63.3) | < 0.005 |
| Unknown | 0 | 5 (2.02) | |
| Operation (gastrectomy) | | | NS |
| Total | 5 (15.6) | 22 (8.87) | |
| Proximal | 0 | 13 (5.24) | |
| Distal | 27 (84.4) | 199 (80.2) | |
| Partial | 0 | 6 (2.42) | |
| Additional resection | 8 (25) | 3 (1.21) | |

C, upper third; M, middle third; A, lower third; I, protruded; IIa, superficial elevated; IIc, superficial depressed; III, excavated; NS, not significant.

**Table II** Histological findings for superficial spreading *vs* small-sized cancers

| *Variable* | *Superficial spreading type (%)* | *Small-sized type (%)* | P-*value* |
|---|---|---|---|
| Depth of invasion | | | < 0.01 |
| Mucosa | 12 (37.5) | 153 (61.7) | |
| Submucosa | 20 (62.5) | 95 (38.3) | |
| Histological type | | | < 0.05 |
| Intestinal | 17 (53.1) | 162 (65.3) | |
| Diffuse | 15 (46.9) | 66 (26.6) | |
| Unknown | 0 | 20 (8.06 | |
| Lymph node metastasis | | | < 0.005 |
| Positive | 7 (21.9) | 7 (2.87) | |
| Negative | 25 (78.1) | 237 (97.1) | |
| Lymph vessel involvement | | | < 0.005 |
| Positive | 5 (15.6) | 9 (3.63) | |
| Negative | 16 (50) | 150 (60.5) | |
| Unknown | 11 (34.4) | 89 (35.9) | |
| Vascular involvement | | | NS |
| Positive | 0 | 6 (2.4) | |
| Negative | 21 (65.6) | 151 (60.9) | |
| Unknown | 11 (34.4) | 91 (36.7) | |
| Cancer–stroma relationship | | | NS |
| Scirrhous | 0 | 3 (1.2) | |
| Intermediate | 11 (34.4) | 61 (24.6) | |
| Medullary | 4 (12.5) | 19 (7.7) | |
| Unknown | 17 (53.1) | 165 (66.5) | |
| Histological growth pattern | | | NS |
| Expansive | 7 (21.9) | 55 (22.2) | |
| Intermediate | 9 (28.1) | 65 (26.2) | |
| Infiltrative | 3 (9.4) | 11 (4.4) | |
| Unknown | 13 (40.6) | 117 (47.2) | |

indistinct tumour margin in eight cases, and distinct tumour margins in seven cases. Of the 15 cases, 11 received an intraoperative histological investigation, but the remaining four cases did not receive it, because the tumour margins were macroscopically obvious. Eight of the 11 cases quickly underwent an additional resection as a continuance of the initial gastrectomy owing to a positive surgical margin found in intraoperative histological examination. The remaining three cases did not receive an additional resection as a result of histologically negative surgical margin. Only three of the 143 patients with small-sized early gastric cancers in the upper two-thirds of the stomach underwent an additional resection after the gastrectomy because of a positive surgical margin. Five patients with superficial spreading lesions (15.6%) underwent a total gastrectomy with a safe surgical margin (more than 3 cm distant from the tumour margin). In contrast, 22 of the 248 patients with small-sized early gastric cancers (8.87%) underwent a total gastrectomy because, although the tumour was located at the gastric cardia, there was no safe surgical margin. Of the five cases of the superficial spreading type who underwent a total gastrectomy, three cases received a total gastrectomy as a continuance of subtotal gastrectomy due to a positive surgical margin. D2 or more extensive lymph node dissection was performed on 29 of the 32 patients with superficial speading lesions (90.6%), and 157 of the 248 patients with small-sized lesions (64.6%). The remaining three cases of the superficial speading type underwent a D1 lymph node dissection because they were older and had operative risk, such as cardiopulmonary complication.

### *Subtypes of superficial spreading lesions*

The superficial spreading type can be divided into two subtypes based on its macroscopic appearance: (1) the elevated subtype (Figure 1) and (2) the depressed subtype

**Figure 1** Elevated subtype of superficial spreading lesions.

**Figure 2** Depressed subtype of superficial spreading lesions.

**Figure 3** Survival curves of patients with superficial spreading lesions and those of patients with small-sized cancers. There was no statistical difference in survival between the two groups.

(Figure 2). The elevated subtype was more common in elderly patients than in younger patients. Submucosal invasion and the intestinal type of cancer were predominantly associated with the elevated subtype. Of the eight patients who underwent an additional resection, seven had the depressed subtype.

### *Survival*

The survival of patients with superficial spreading lesions was compared with patients with small-sized lesions. Patients who died of other diseases, including post-operative complications, were excluded from this analysis. There was no significant difference in the 5 year survival rates of the two groups (Figure 3, superficial spreading type, 96.0% *vs* small-sized type, 95.1%). Of the 32 patients with superficial spreading lesions, one and three died of liver metastasis and other diseases respectively. Of the 248 patients with small-sized lesions, four and nine died of recurrent gastric cancer and other diseases respectively. Operative death was observed in two patients with small-sized early gastric cancers, but not in patients with superficial spreading lesion. Post-operative complications, such as pneumonia or anastomotic leakage, occurred in both groups, but its frequency did not differ between the two groups.

### Discussion

The majority of gastric cancers, as well as other solid gastrointestinal cancers, usually grow along and deeply into the stomach wall in a three-dimensional manner. It is very surprising and interesting to note that the superficial spreading type of gastric cancer spreads superficially along the stomach wall, but it does not penetrate into the stomach wall. Thus, this type of cancer is not just a slightly more advanced degree of early gastric cancer; the superficial spreading type of early gastric cancer should be considered as a gastric cancer variant, which possesses a unique feature different from other types of gastric cancer.

The superficial spreading type of early gastric cancer demonstrated histological aggressiveness, including submucosal invasion, a diffuse type and advanced invasion into the lymphatic system. Gastric cancers with these histological findings have generally been believed to show a poor prognosis compared with those without such histological aggressiveness. This belief is based on the following observations, which have been previously reported: (1) patients with submucosal invasion apparently show a poorer prognosis than those with only mucosal invasion (Maehara *et al.*, 1993); (2) the diffuse type is more likely to infiltrate the surrounding tissues and often produces large and invasive tumours, resulting in dismal prognostic results (Maehara *et al.*, 1991); and (3) the involved lymphatic systems are important factors which affect the prognosis of gastric cancer (Maruyama *et al.*, 1987). However, the histological aggressiveness did not actually affect the prognosis of patients with superficial spreading lesions. These results are attributable probably to the extended surgical treatment, consisting of a wide resection of the stomach with extensive lymph node dissection. If we had performed limited surgical treatment for superficial spreading lesions, then gastric cancer remnants or lymph node recurrence might occur. Thus, limited surgery for small-sized early gastric cancers is not indicated for superficial spreading types of lesions.

Another explanation for the discrepancy between the prognosis and the histological aggressiveness of the superficial spreading type is the rarity of cancer death caused by distant metastasis. Death as a result of recurrent gastric cancer was found in only one of 32 patients. This suggests that this type of cancer may possess a biologically more mild nature with respect to distant metastasis. This type of cancer also spreads widely and superficially along the mucosal layer of the stomach, but does not produce the distant metastases, which are responsible for the prognosis of early gastric cancers.

To explain the biologically mild behaviour of the superficial spreading type, one interesting study described the low expression of epidermal growth factor (EGF) and transforming growth factor (TGF) in superficial spreading lesions (Hirayama *et al.*, 1990). Since EGF and TGF have been reported to be connected with tumour growth and invasion (Sugiyama *et al.*, 1989), the low expression of these factors in superficial speading lesions may underlie its biologically mild behaviour. Thus, additional chemotherapy to prevent distant metastases should not be necessary for this type of lesion. In other words, surgery alone can erradicate this type of cancer, and can lead to a complete cure.

The superficial spreading type of cancer was divided into the elevated and depressed subtypes in this study. There were three main differences in the clinicopathological findings between these two subgroups relating to: (1) the depth of invasion; (2) the histological type; and (3) the tumour margin.

Submucosal invasion was predominant for the elevated subtype of superficial spreading cancer, and this observation was obviously in contrast to the elevated lesions of small-sized early gastric cancers. The intestinal type was predominant in the elevated subtype of superficial spreading cancer, which was similar to the findings for elevated lesions in small-sized early gastric cancers. The predominance of the intestinal type in elevated lesions is common to all types of gastric cancer, as reported previously (Correa, 1984).

The most interesting difference between the two subtypes was in their surgical treatment; the depressed subtype required an additional resection after gastrectomy more frequently than did the elevated subtype. One important factor in the surgical treatment of gastric cancers is the extent of the resection, i.e. a partial, subtotal or total gastrectomy.

To determine the resection lines, we must accurately define the tumour margin because, otherwise, all of the cases must undergo a total gastrectomy. The tumour margins of the depressed lesions were so indistinct that the resection lines were occassionally difficult to determine. As a result, additional resections after a gastrectomy or a total gastrectomy were required more often for the depressed lesions. We can now present an excellent method for avoiding unnecessary resections or cancer remnants in the stomach: contrast endoscopy using dye-spraying techniques (Richart, 1963).

In conclusion, the superficial spreading type of early gastric cancer should be considered as a disease entity that is different from other types of early gastric cancers. Its characteristics are submucosal invasion, a diffuse type, advanced lymph node involvement, and an indistinct tumour margin. The most appropriate treatment for the superficial spreading type should be a wide resection with extensive lymph node dissection.

### References

CORREA P. (1984). Pathology of gastric cancer. *Clin. Oncol.*, **3,** 251–257.

HEESAKKER JPFA, GOUMA DJ, THUNNISSEN FBJM, BEMELMANS MHA AND VON MEYENFELDT MF. (1994). Non-radical therapy for early gastric cancer. *Br. J. Surg.*, **81,** 551–553.

HIRAYAMA K, FUJIMORI T, MOTOTSUGU A AND MAEDA S. (1990). Clinicopathological and immunohistochemical study on penetrating and superficial spreading type of early gastric cancers. *Jpn. J. Gastroenterol.*, **87,** 2434–2443.

ICHIYOSHI Y, TODA T, MINAMISONO Y, NAGASAKI S, YAKEISHI Y AND SUGIMACHI K. (1990). Recurrence of early gastric cancer. *Surgery*, **107,** 489–495.

JAPANESE RESEARCH SOCIETY FOR GASTRIC CANCER. (1981). The general rules for the gastric cancer study in surgery and pathology. Part II. *Jpn. J. Surg.*, **11,** 140–145.

LAUREN P. (1965). The two histological main types of gastric carcinoma: diffuse and so-called intestinal type carcinoma. *Acta Pathol. Microbiol. Immunol. Scand.*, **64,** 31–49.

MAEHARA Y, MORIGUCHI S, KAKEJI Y, ORITA H, HARAGUCHI M, KORENAGA D AND SUGIMACHI K. (1991). Prognostic factors in adenocarcinoma in the upper third of the stomach. *Surg. Gynecol. Obstet.*, **173,** 223–226.

MAEHARA Y, OKUYAMA T, OSHIRO T, BABA H, ANAI H, AKAZAWA K AND SUGIMACHI K. (1993). Early carcinoma of the stomach. *Surg. Gynecol. Obstet.*, **177,** 593–597.

MARUYAMA K, OKABAYASHI K AND KINOSHITA T. (1987). Progress in gastric cancer in Japan and its limits of radicality. *World J. Surg.*, **11,** 418–425.

RICHART RM. (1963). A clinical staining test for the *in vivo* delineation of dysplasia and carcinoma *in situ*. *Am. J. Obstet. Gynecol.*, **86,** 703–712.

SANO T, KOBORI O AND MUTO T. (1992). Lymph node metastasis from early gastric cancer: endoscopic resection of tumor. *Br. J. Surg.*, **79,** 241–244.

SOWA M, YASUUKI K AND MASANORI N. (1991). Surgical approach to early gastric cancer with lymph node metastasis. *World J. Surg.*, **13,** 630–636.

STOUT AP. (1942). Superficial spreading type of carcinoma of the stomach. *Arch. Surg.*, **44,** 651–657.

SUGIYAMA K, YONEMURA Y AND MIYAZAKI I. (1989). Immunohistochemical study of epidermal growth factor and epidermal growth factor receptor in gastric carcinoma. *Cancer*, **63,** 1557–1561.

YASUI A, HIRASE Y, MIYAKE M, KIDOKORO T AND MURAKAMI T. (1973). Pathology of superficial spreading type of gastric cancer. *I To Cho*, **8,** 1305–1310 (in Japanese).